# Oral Lesions: The Clue to Diagnosis of Pemphigus Vulgaris

**DOI:** 10.1155/2015/593940

**Published:** 2015-11-19

**Authors:** Diana Kuriachan, Rakesh Suresh, Mahija Janardhanan, Vindhya Savithri

**Affiliations:** Department of Oral Pathology and Microbiology, Amrita School of Dentistry, Amrita Vishwa Vidyapeetham, Kochi 682041, India

## Abstract

Pemphigus is a group of potentially fatal dermatoses with both cutaneous and oral manifestations. Characterized by the appearance of vesicle or bullae, their manifestations in the oral cavity often precede those on the skin by many months or may remain as the only symptoms of the disease. It is therefore important that the oral manifestations of the disease are recognized on time, to make a proper diagnosis and initiate timely treatment. Here we present a case of Pemphigus Vulgaris (PV) that presented with oral lesions at multiple sites including tongue, to highlight the importance of timely recognition of the oral lesions during routine dental practice for the diagnosis and management of this disease.

## 1. Introduction

Pemphigus Vulgaris (PV) is the most common variant of the Pemphigus group of potentially fatal autoimmune diseases characterized by cutaneous or mucosal blistering and shows oral lesions as early manifestations of the disease in nearly 50% of the cases [1, 2]. Its peak incidence is between the fourth and fifth decade of life [3]. Clinically oral lesions precede skin lesions in many cases and appear as blisters which rupture rapidly resulting in painful erosions. Buccal mucosa, lips, and soft palate are most commonly involved [4]. Diagnosis is based on the identification of clinical manifestations and confirmation through biopsy. Demonstration of immunoglobulins, in the spinous cell junctions by distinct immunofluorescence (IF), is often used for the final confirmation of PV [5, 6]. As the oral presentation of the disease is often the first indicator that can lead to the final diagnosis, it is very critical for the dental practitioner to recognize the oral lesions of PV at a sufficiently early stage to initiate further investigations and treatment. We present a case of PV where the patient presented with ulcerations at multiple oral sites including tongue and the final diagnosis was made by the timely interpretation of these manifestations.

## 2. Case Report of Pemphigus Vulgaris at Multiple Intraoral Sites, with No Involvement of Skin

**Figure 1:**
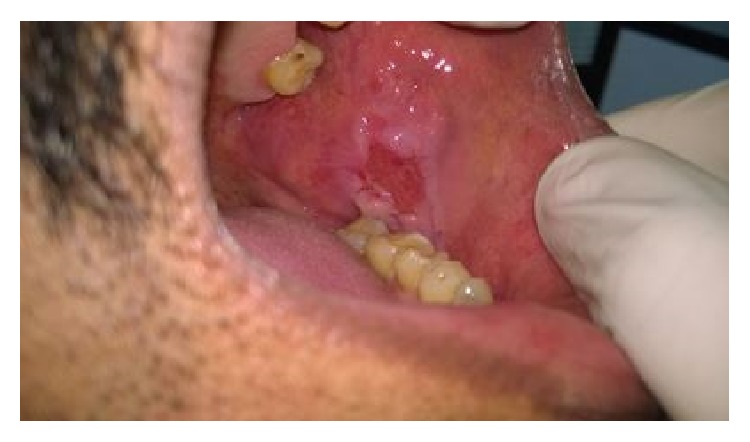
Ulcer on the left buccal mucosa, ovoid in shape.

**Figure 2:**
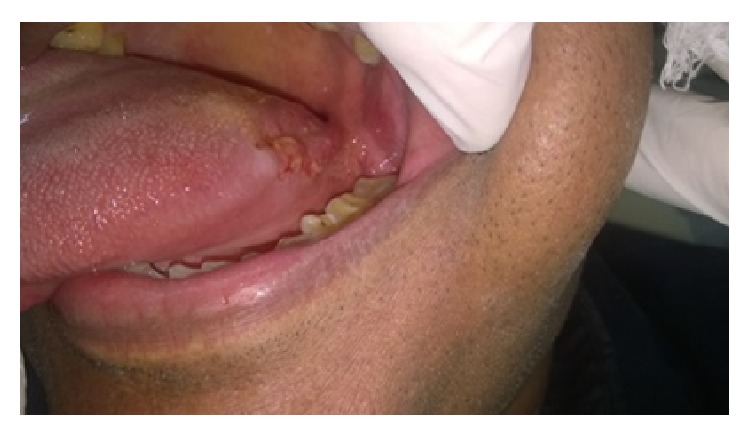
Ulcer with yellow crusted surface on the left posterolateral border of tongue.

**Figure 3:**
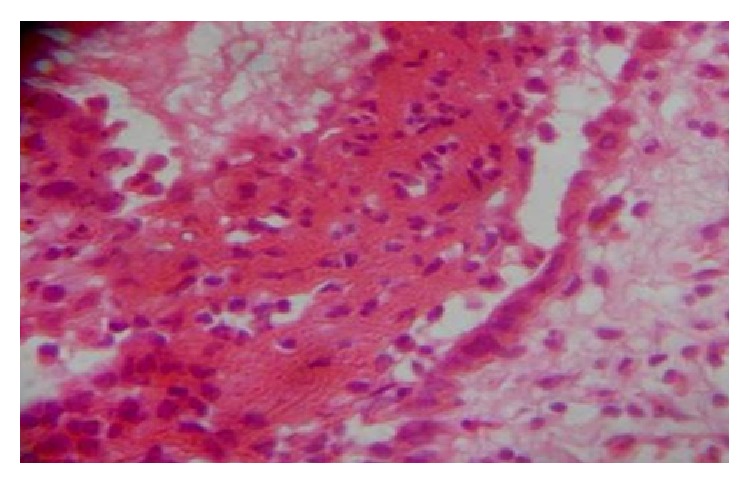
Epithelium exhibiting suprabasal split (H&E stain, ×100).

**Figure 4:**
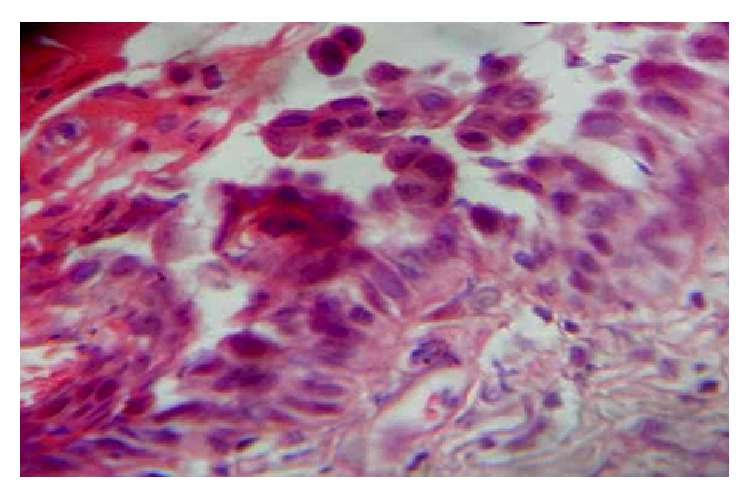
Acantholytic Tzanck cells within the suprabasal split (H&E stain, ×400).

A 55-year-old gentleman presented with painful nonhealing ulcers on the left buccal mucosa and left posterolateral border of tongue four months ago. History revealed that he had burning sensation at both sites for the past six months. He was aware of one blister which appeared and burst rapidly on the buccal mucosa, after which ulcerations appeared on both the sites. There was no history of skin lesions. Intraoral examination revealed a 2 cm × 2 cm ovoid shallow ulcer with sloping margins along the line of occlusion of 35 to 37 on the left buccal mucosa (Figure 1) and a 1 cm × 1 cm ovoid ulcer with yellow crusted surface on the left posterolateral border of the tongue (Figure 2). After ascertaining the absence of traumatic agents like sharp tooth/cusp, dentures, and so forth, a provisional diagnosis of vesiculobullous lesions, namely, Pemphigus, Pemphigoid, or Bullous Lichen Planus, was considered. Incisional biopsy was performed and adequate tissue bits were taken from both sites for histopathologic examination. Bits from the perilesional area were also sent for direct IF studies separately. Histopathologic features of the sections from both the sites were similar and showed ulcerated stratified squamous epithelium exhibiting suprabasal split (Figure 3). Many round acantholytic (Tzanck) cells with hyperchromatic nuclei were observed within the split (Figure 4). Basal cells were seen attached to the underlying connective tissue, below the split. A dense inflammatory cell infiltrate consisting mainly of plasma cells was seen in the connective tissue. These microscopic features were suggestive of PV. The direct IF showed deposits of IgG and C3 (complement) in a fish-net pattern along the spinous intercellular zone, which confirmed the diagnosis of PV.

## 3. Discussion

Derived from the Greek word meaning “blister,” Pemphigus is a group of potentially life-threatening autoimmune mucocutaneous disorders characterized by intraepithelial blister formation [1]. The blisters occur in the epithelium where the patients IgG autoantibodies produced in response to triggering factors target two structured proteins of desmosomes identified as Desmogleins 1 and 3. Recently, a new Pemphigus antigen Desmoglein 4 and other non-Desmoglein antigens like human *α*-9-acetylcholine receptor that regulates keratinocyte adhesion and keratinocyte annexin like molecules binding acetylcholine termed pemphaxin and catenin are also thought to play a role in its etiopathogenesis [7, 8]. Thin separation at the desmosomal region triggers the acantholysis and suprabasal spilt.

Pemphigus usually affects patients in the fourth and fifth decades of life, with females reportedly being affected more frequently than males [3]. More than 50% of the affected patients have been reported with initial manifestations on the oral mucosa followed by skin involvement. The average duration of the oral lesions is found to be between 3 months and one year [9].

Our patient presented with oral lesions in the form of ulcerations four months ago and was aware of one blister forming prior to this. As the oral cavity is subject to trauma during mastication, the thin roof of the blister ruptures easily and forms an erosion or ulcer in the area. This patient did not develop any lesion on the skin [10]. As reported in literature our patient too presented with the two most common symptoms related to PV, that is, pain and burning sensation. Many cases of PV have been reported to begin as generalized lesions involving multiple intraoral sites as in our case where the patient developed lesions on the buccal mucosa and the tongue. Though buccal mucosa has been reported to be one of the most commonly affected sites, tongue, which was also affected in our case, is a rare site for PV [9]. Since the clinical features of PV are similar to those seen in Cicatricial Pemphigoid and Bullous Lichen Planus, its diagnosis needs to be confirmed with routine histopathology and IF studies. Tissue bits from both sites were taken in our case and the histologic features were suggestive of PV. The diagnostic features were the presence of a suprabasilar split and acantholytic Tzanck cells in the split, both produced due to the intraepithelial blister formation. Direct IF study showed the typical “fish-net” pattern of IgG and complement C3 deposits in the spinous layer, the site for the autoimmune reaction in PV. Both histopathology and IF studies confirmed the diagnosis of PV.

PV is generally treated with oral, intralesional, and topical corticosteroids [11]. The present treatment regime in PV is based on systemic immunosuppressant like corticosteroids along with adjuvants like methotrexate, cyclophosphamide, and so forth [6]. Drugs like cholinergic agonists are thought to reverse the acantholysis in PV [5]. Our patient, in consultation with the department of dermatology, was put on 100 mg dexamethasone for 3 days along with 500 mg of cyclophosphamide. Two more cycles of this regime at intervals of 4 weeks each are planned. The patient was put on 30 mg Wysolone tablets during the interim 4-week period. A review after 2 weeks of the initial steroid therapy showed that the cheek and the tongue ulcerations were resolving which indicated a positive response to the treatment.

PV, a potentially fatal disease with most cases showing initial oral manifestations, requires early diagnosis as well as early treatment to prevent future complications. The diagnosis of PV is based on 3 main factors, clinical features, histopathology, and immunofluorescence studies. Many a time, keen observation of the oral symptoms leading to a histopathologic examination will suffice for a final diagnosis. This in turn can facilitate early treatment which will be highly beneficial to the patient's recovery. Nevertheless, long term regular follow-up is essential to identify the possible remissions of this disease.
